# Urbanization Breaks Up Host-Parasite Interactions: A Case Study on Parasite Community Ecology of Rufous-Bellied Thrushes (*Turdus rufiventris*) along a Rural-Urban Gradient

**DOI:** 10.1371/journal.pone.0103144

**Published:** 2014-07-28

**Authors:** Cláudia Calegaro-Marques, Suzana B. Amato

**Affiliations:** Departamento de Zoologia, Instituto de Biociências, Universidade Federal do Rio Grande do Sul, Porto Alegre, Rio Grande do Sul, Brazil; Behavioural Ecology & Ecophysiology group, Denmark

## Abstract

Urbanization drastically alters natural ecosystems and the structure of their plant and animal communities. Whereas some species cope successfully with these environmental changes, others may go extinct. In the case of parasite communities, the expansion of urban areas has a critical effect by changing the availability of suitable substrates for the eggs or free-larval stages of those species with direct life cycles or for the range of hosts of those species with complex cycles. In this study we investigated the influence of the degree of urbanization and environmental heterogeneity on helminth richness, abundance and community structure of rufous-bellied thrushes (*Turdus rufiventris*) along a rural-urban gradient in the metropolitan region of Porto Alegre, State of Rio Grande do Sul, Brazil. This common native bird species of southern Brazil hosts 15 endoparasite species at the study region. A total of 144 thrushes were collected with mist nets at 11 sites. The degree of urbanization and environmental heterogeneity were estimated by quantifying five landscape elements: buildings, woodlands, fields, bare lands, and water. Landscape analyses were performed at two spatial scales (10 and 100 ha) taking into account home range size and the potential dispersal distance of thrushes and their prey (intermediate hosts). Mean parasite richness showed an inverse relationship with the degree of urbanization, but a positive relationship with environmental heterogeneity. Changes in the structure of component communities along the rural-urban gradient resulted from responses to the availability of particular landscape elements that are compatible with the parasites' life cycles. We found that the replacement of natural environments with buildings breaks up host-parasite interactions, whereas a higher environmental (substrate) diversity allows the survival of a wider range of intermediate hosts and vectors and their associated parasites.

## Introduction

Human population growth and rural exodus have jointly caused a rapid expansion of urban areas throughout the world in the past century [1]. Urbanization is an extreme form of environmental alteration that often leads to a complete restructuring of plant communities [2]. As a consequence, the richness and abundance of animals are also radically altered in urban landscapes [3]–[5].

Although some species are successful in coping with these drastic anthropogenic disturbances in their native habitat and adapt to (or even thrive in) the urban landscape [6], many (probably most) of them are less tolerant and may go locally extinct [4]. These trends were witnessed in all major taxonomic groups so far studied (mammals, birds, reptiles, amphibians, invertebrates and plants [7]), among which birds are the best known examples [8]–[11]. In a comparative review Marzluff [3] found evidence of a decrease in species richness in more urbanized environments in 31 out of 51 (61%) bird studies along urban-rural gradients. Exceptions have been described in areas with moderate degrees of urbanization, where elements of the native biota coexist with non-invasive species introduced by humans, especially exotic plants used in gardening and landscaping. McKinney [7] reports that 65% of studies showed an increase in plant richness in these moderately urbanized areas, but the figures for invertebrates and vertebrates were much lower (30% and 12%, respectively).

In addition to the aforementioned influence on plant-animal interactions, the environmental changes found along the urban-rural gradient that goes from the core of totally built-up metropolitan areas to their semi-natural surroundings and more remote regions or least altered ecosystems [4], [12] may represent a considerable challenge for parasite species. Although parasites whose transmission occurs via active skin penetration or the ingestion of eggs and larvae also require a suitable substrate for their survival, urbanization is particularly critical for those species whose successful development demands the interaction with multiple hosts during their complex life cycles. Parasites may have important influences in their ecosystems, may play a key role in species conservation [13], and provide services to humans (*e.g.*, sentinels for environmental pollutants and other health-related benefits [14]–[17]). Therefore, the disappearance or reduction in abundance of host species may create a gap in parasite cycles, leading to a decrease in their transmission capacity [18].

Birds are important hosts for a wide variety of helminth parasites, including digeneans, cestodes, nematodes and acanthocephalans [19], but most research on the diversity of parasites in urban birds have focused on viruses, bacteria, protists, and ectoparasites (reviewed by Delgado-V. and French [20]). Only a handful of studies have addressed the helminth endoparasites of urban avifauna: *Columba livia* in Brazil [21], Chile [22] and Spain [23]; *Columbina picui* [24], *Passer domesticus* [25] and *Turdus rufiventris* [26], [27] in Brazil. In addition, no research has addressed the spatial relationships between urban bird hosts and their associated helminthfauna along a rural-urban gradient [20].

This research begins to fill this gap by investigating the influence of the degree of urbanization and environmental heterogeneity on the richness, abundance and community structure of helminth parasites of the rufous-bellied thrush (*Turdus rufiventris*) in a rural-urban gradient in sub-tropical southern Brazil. The following four hypotheses and their respective predictions are tested: (1) if urbanization reduces the diversity of helminth parasites by replacing suitable habitats for intermediate hosts and vectors with unsuitable built-up areas, then the proportion of built-up areas will show an inverse relationship with parasite species richness; (2) if environmental heterogeneity has a positive effect on the diversity of helminth parasites by providing a variety of suitable habitats for intermediate hosts and vectors, then the diversity of landscape elements will show a direct relationship with parasite species richness; (3) if the structure of helminth parasite communities differs along a rural-urban gradient because of the environmental changes resulting from urbanization, then the degree of urbanization will be a good predictor of spatial differences in parasite community structure; (4) if parasites respond to the rural-urban gradient in a direction compatible with their life cycle and the habitat requirements of their intermediate hosts or vectors, then individual species will respond differently to the proportion of particular landscape elements.

*Turdus rufiventris* is a good model to apply this approach because it is a generalist faunivore-frugivore bird that is well-adapted to the city environment [26], [28], [29] and is a common native resident species throughout the rural-urban gradient of the study region [11], [30]. Thrushes (*Turdus* spp.) may also show a high site fidelity to their small home ranges of less than 2 ha [31], [32]. Additionally, 13 of the 15 helminth parasites of *T. rufiventris* have complex life cycles that involve intermediate invertebrate hosts (the digeneans *Brachylaima* sp., *Conspicuum conspicuum*, *Lutztrema obliquum* and *Tamerlania inopina*; the cestodes *Dilepis undula*, *Fernandezia spinosissima* and *Wardium fernandensis*; the acanthocephalan *Lueheia inscripta*; and the nematodes *Aonchoteca* sp., *Microtetrameres pusilla* and *Oxyspirura petrowi*) or mosquito vectors (the nematodes *Aproctella stoddardi* and *Cardiofilaria* sp.). Two species, the nematodes *Strongyloides oswaldoi* and *Syngamus trachea*, have monoxen life cycles, but the latter may involve a paratenic host [26].

## Methods

Rufous-bellied thrushes (N = 144) were collected using mist nets in 11 sites (30^°^07'54''S, 51^°^03'46''W; 30^°^05'55''S, 51^°^10'26''W; 30^°^04'15''S, 50^°^01'20''W; 30^°^06'10''S, 51^°^12'47''W; 30^°^09'00''S, 50^°^53'43''W; 30^°^00'45''S, 51^°^06'36''W; 30^°^01'38''S, 51^°^11'57''W; 30^°^08'24''S, 50^°^52'00''W; 30^°^01'50''S, 51^°^13'08''W; 30^°^01'11''S, 51^°^11'20''W; 30^°^03'41''S, 51^°^10'35''W) in a rural-urban gradient in the metropolitan region of Porto Alegre, State of Rio Grande do Sul, Brazil, between March 2003 and March 2006. Location sites were authorized by the local environmental authority (Secretaria Municipal do Meio Ambiente/SMAM, permits *#*176/04 and 049/05), whereas the trapping and transport were authorized by the Brazilian Environmental Protection Agency (Instituto Brasileiro do Meio Ambiente e dos Recursos Naturais Renováveis/IBAMA, permits *#*051/2002/RS, 005/2004/RS and 004/2005/RS). *Turdus rufiventris* is a South American species listed as Least Concern by IUCN [33] and is not listed under any threatened category in the Red Lists of Brazil [34] and the State of Rio Grande do Sul [35].

Birds were killed with an overdose of gaseous anesthetic following the guidelines of the American Ornithological Council [36]. The research protocol was approved by the Biological Sciences Research Committee (Comissão de Pesquisa em Ciências Biológicas – Pró-Reitoria de Pesquisa) of the Universidade Federal do Rio Grande do Sul (Project #8468, approved in 16 March 2004). Thrush necropsy and helminth processing followed standard procedures [37]. Carcasses were deposited at the Museu de Ciências e Tecnologia of the Pontifícia Universidade Católica do Rio Grande do Sul (MCT/PUCRS) and voucher helminth specimens were deposited at the Coleção Helmintológica of the Instituto Oswaldo Cruz (CHIOC), Rio de Janeiro, Brazil (#35672 – 35677, 36508, 37266, 37267, 37269 – 37273).

Mean parasite richness and mean abundance of each taxon in each study site were calculated to reduce possible effects of differences in sample sizes (range: 4 to 35; mean ± s.d.  = 13±11). Regression analyses showed that sample size per site had no significant relationship with these variables. Therefore, community structure was estimated based on the mean abundance of each helminth taxon in each site.

The degree of urbanization and the environmental heterogeneity of each site were estimated by quantifying the proportion of five landscape elements:

- built-up areas with buildings, streets and roads (hereafter buildings);

- wooded areas, such as small patches of trees, woodlands or forests (hereafter woodlands);

- grass-covered surfaces, including lawns and natural fields (hereafter fields);

- bare lands, including fallows and degraded soil (hereafter bare lands);

- water surfaces, such as springs, lakes, ponds, and mill-dams (hereafter water).

This quantification was obtained by employing processing routines and orbital imaging using the software Idrisi Andes 15.0, Windows platform. The analyzed orbital image was acquired on 31 January 2003 by the sensor Landsat ETM 7+, orbital point 221/081, bounding coordinates UTM 467230m, 515020mE; 6661430m, 6700220mN. Three spectral bands (red, near infrared and mid-infrared) were used for compiling the RGB color composite system. Geometric and radiometric corrections, unsupervised classification and clustering method were also performed. Pixels (resolution 30×30 m) in the raster image were classified and clustered based on their Euclidean distances. Similar methodology has been applied in studies focusing on the effect of urbanization on bird ecology and conservation [38], [39].

**Figure 1:**
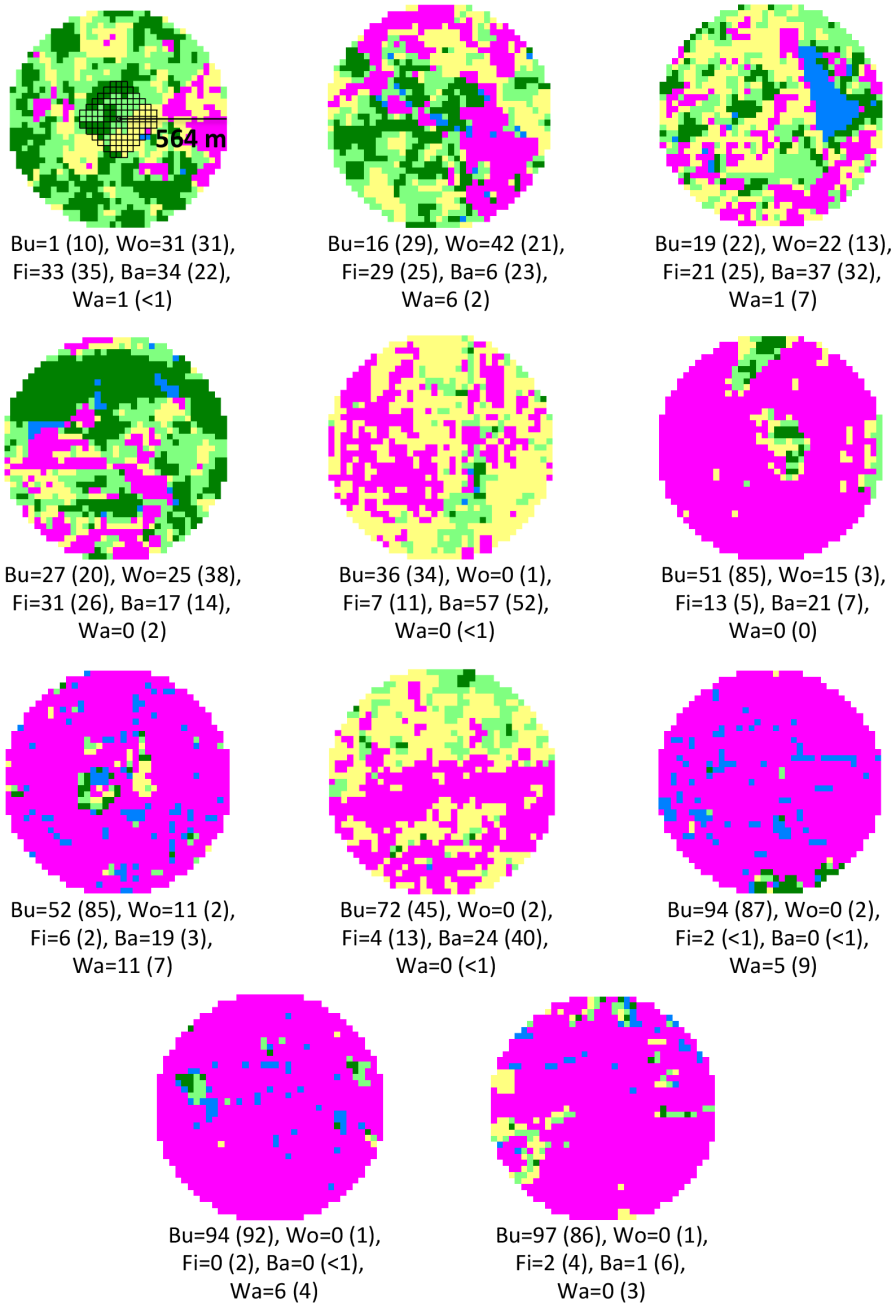
Sketch of the study sites showing the representation and distribution of each landscape element. Sites are organized from left to right and from top to bottom in increasing order of the representation (%) of buildings at the 10-ha scale (see grid in the first site). The representation of each landscape element at the 100-ha scale is shown in parentheses. Buildings (Bu, pink), woodlands (Wo, dark green), fields (Fi, light green), bare lands (Ba, yellow), and water (Wa, blue).

The landscape analysis was performed at two spatial scales (10- and 100-ha) around each collection site (Figure 1). These two scales were chosen taking into account the potential dispersal of thrushes and their prey – the intermediate hosts. The 10-ha area is about five-fold the size of the larger home ranges reported for *Turdus* spp. and shall cover the maximum dispersal distance of intermediate hosts, particularly those with limited mobility, such as annelids, mollusks, millipeds, isopods, and some insects. The more conservative scale of 100 ha was chosen because there are no data on dispersal distance, home range size, day range length, and site fidelity for *T. rufiventris* and because some intermediate hosts, particularly flying insects, may disperse over larger areas. The percentage of buildings was used as a proxy of the degree of urbanization, and the percentages of each landscape element were used to estimate landscape heterogeneity by the Shannon index of diversity (H'; [40]).

The relationship between mean parasite species richness per host (dependent variable) and both the proportion of built-up areas (hypothesis 1) and the H' of landscape elements (hypothesis 2) - independent variables - was tested using one-tailed regression analyses. Nonmetric multidimensional scaling (NMDS) was used to ordinate the 11 helminth parasite communities based on the Bray-Curtis similarity index to test whether their structures differ along the rural-urban gradient. The influence of urbanization on the NMDS community ordination was tested by the Spearman rank correlation between % buildings and the generated gradients of similarity along the axes (hypothesis 3). Pairwise comparisons were applied to determine the Bray-Curtis similarity percentage (SIMPER) between component communities and the contribution of each helminth taxon to the overall pattern of similarity. Stepwise regression with forward selection was used to evaluate whether any or a combination of landscape elements was a good predictor of the variance in the abundance of each helminth species (hypothesis 4). Regression analyses and Spearman rank correlations were run in BioEstat 5.0 [41] and NMDS and SIMPER were run in PAST [42]. Statistical significance was established at P ≤ 0.05.

## Results

**Figure 2:**
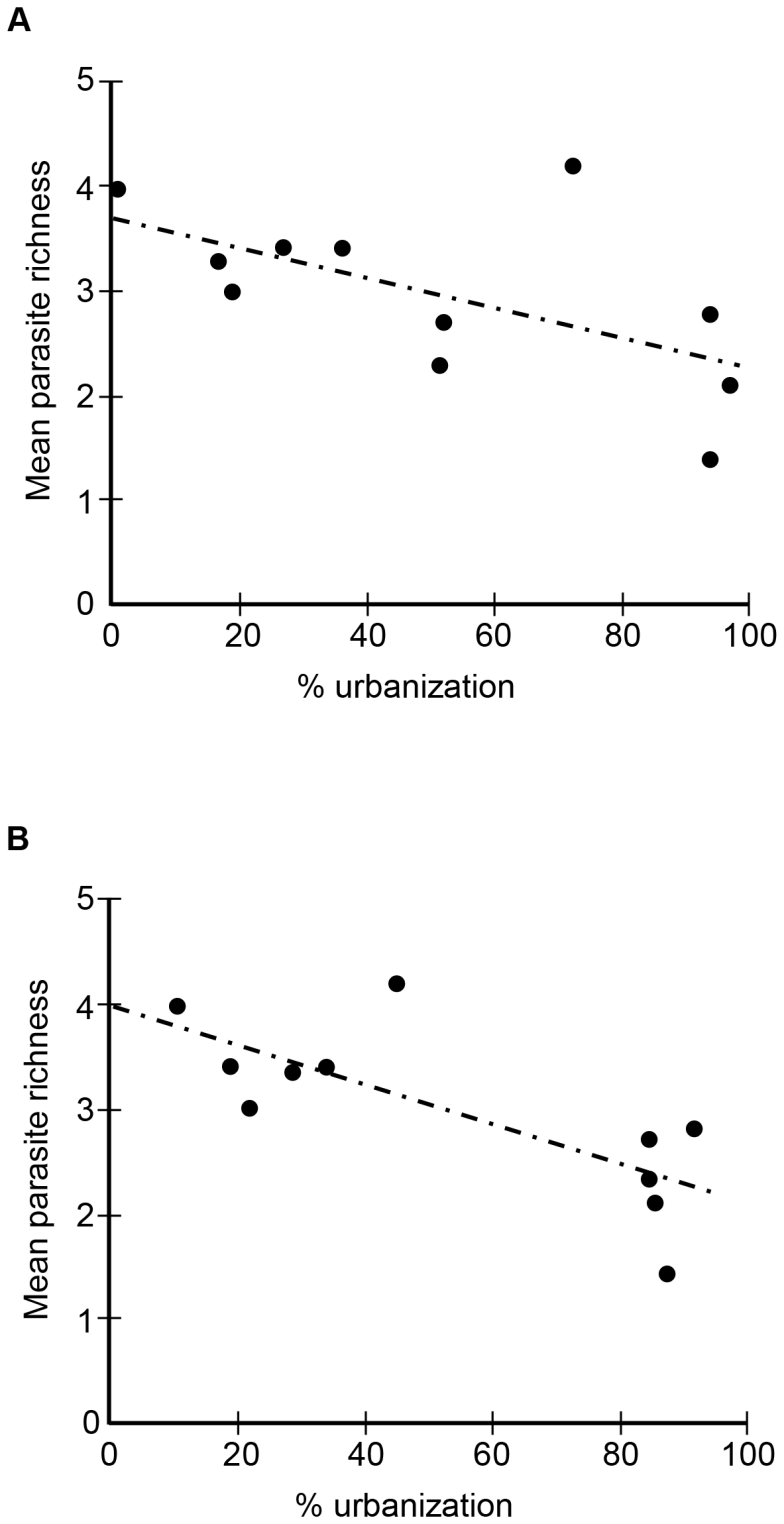
Linear regression between % urbanization (independent variable) and mean parasite richness per host (dependent variable) at the (A) 10-ha and (B) 100-ha scales.

**Figure 3:**
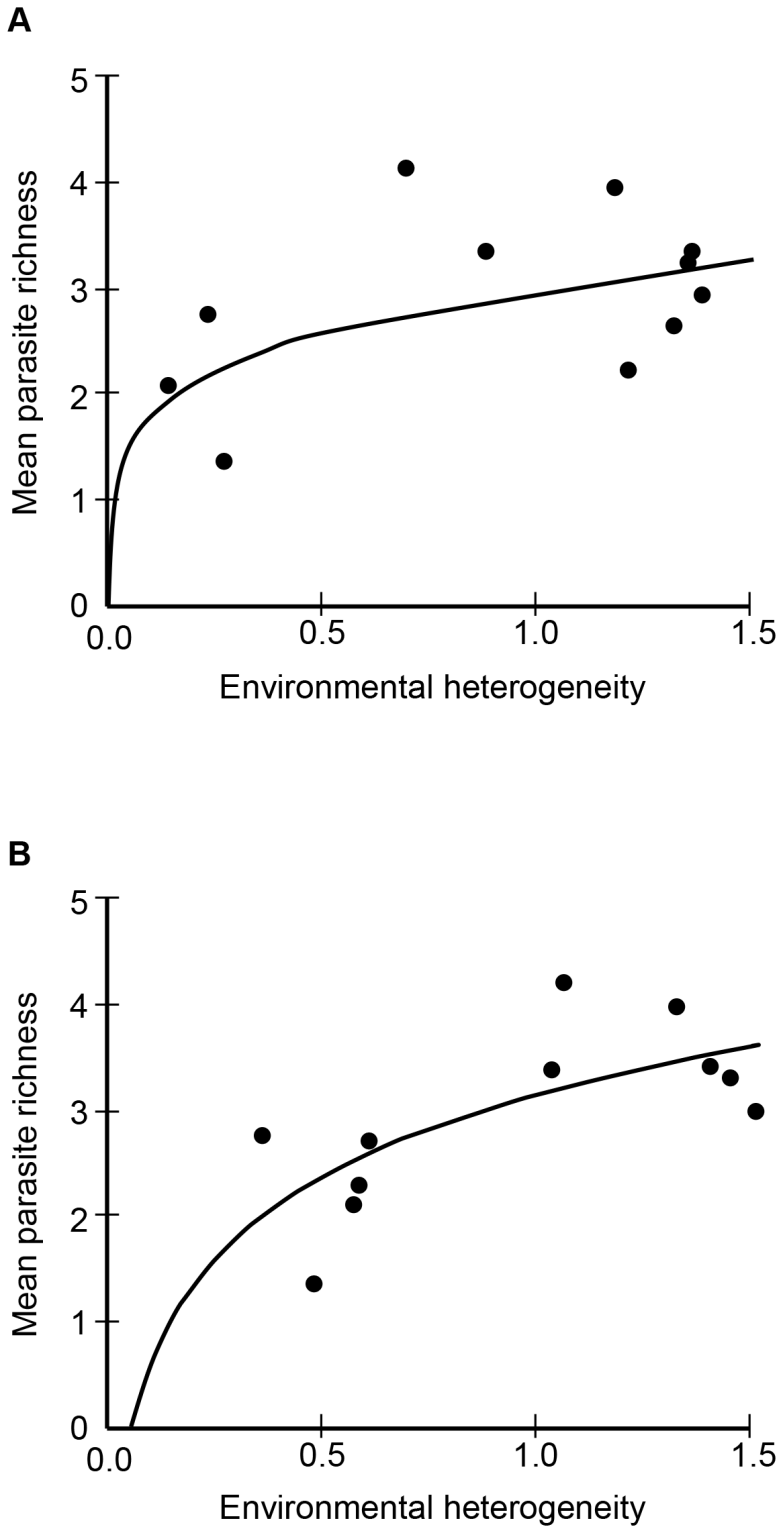
Relationship between environmental heterogeneity (H') and mean parasite richness at the (A) 10-ha and (B) 100-ha scales.

Parasite species richness per site ranged from 4 to 13 (mean ± s.d.  = 9±3, N = 11) and mean richness per host per site ranged from 1.4 to 4.2 (mean ± s.d.  = 3.0±0.8, N = 11). The degree of urbanization (% buildings) showed an inverse relationship with mean parasite richness at both spatial scales (10 ha: r^2^ = 0.357, F = 5.014, DF = 1, p = 0.025, Figure 2a; 100 ha: r^2^ = 0.571, F = 11.986, DF = 1, p = 0.0036, Figure 2b), supporting hypothesis 1. Environmental heterogeneity (H') showed positive relationships with mean parasite richness at the 10-ha (geometric: r^2^ = 0.316, F = 4.170, DF = 1, p = 0.036, Figure 3a) and the 100-ha (logarithmic: r^2^ = 0.490, F = 8.670, DF = 1, p = 0.008, Figure 3b; linear: r^2^ = 0.465, F = 7.810, DF = 1, p = 0.0104) scales, supporting hypothesis 2. As shown in the figures, the curves that best fit the data showed a steeper increase of the dependent variable (helminth diversity) at the lower range of the predictor variable (habitat heterogeneity). However, it is important to note that the geometric model explained less than one third of the variation in the data at the 10-ha scale, whereas the logarithmic and linear models explained about half of it at the 100-ha.

**Figure 4:**
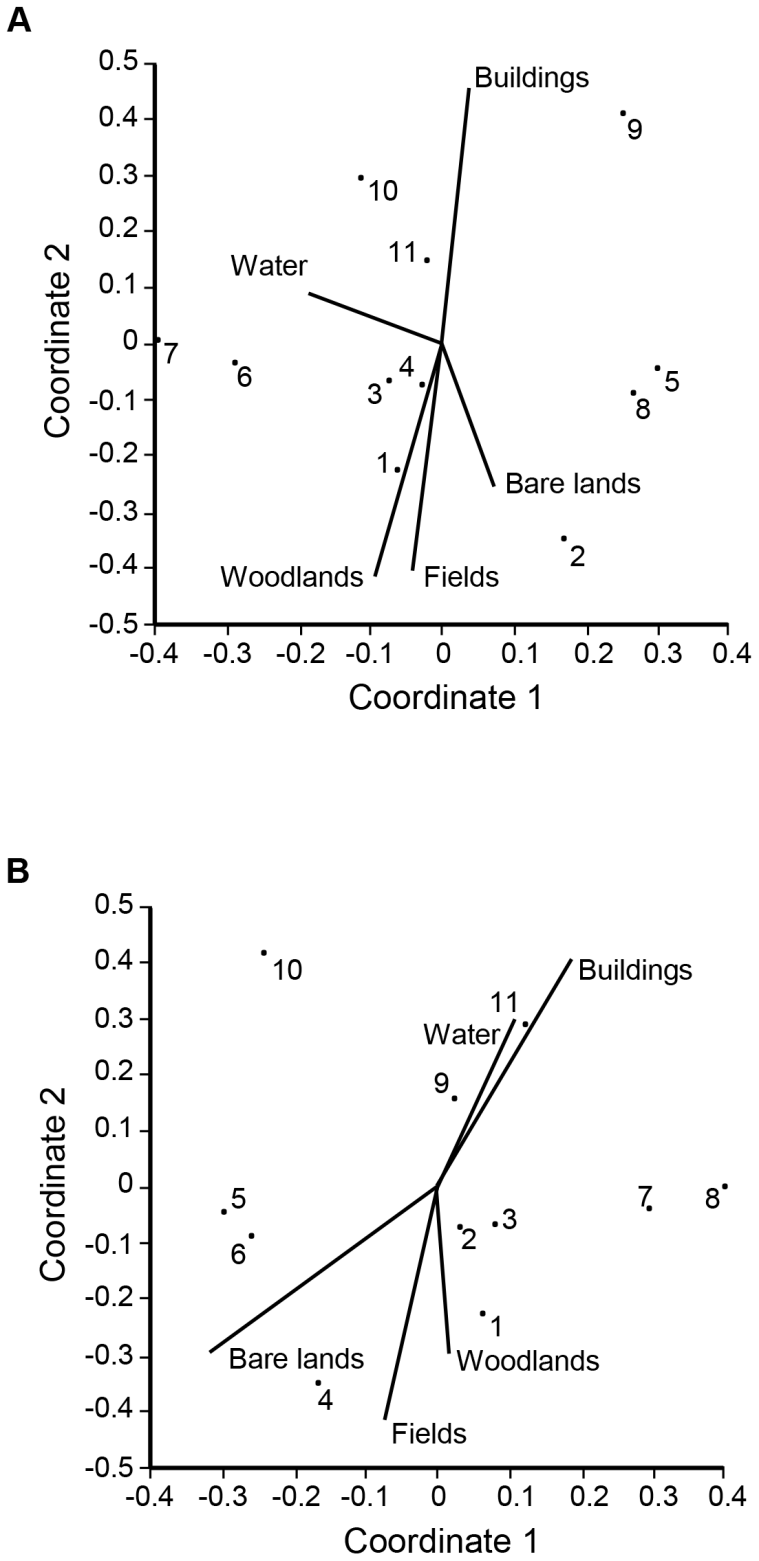
The influence of urbanization on the NMDS community ordination at the (a) 10-ha (stress = 0.1536) and (b) 100-ha (stress = 0.1544) scales.

The structure of component communities varied along the rural-urban gradient at both scales (stress = 0.154). Five species (*S. oswaldoi*, *M. pusilla*, *W. fernandensis*, *S. trachea* and *C. conspicuum*) were responsible for 2/3 of the pooled overall dissimilarity of 61.3% between communities (Table 1). The degree of urbanization had no significant effect on community ordination along axis 1 (10-ha: r_s_ = 0.054, p = 0.873; 100-ha: r_s_ = 0.109, p = 0.749), but it had a strong influence on the ordination along axis 2 at both scales (10-ha: r_s_ = 0.819, p = 0.002; 100-ha: r_s_ = 0.863, p = 0.0006) (Figures 4a e 4b). Hypothesis 3 is also supported.

**Table 1:** Average pair-wise dissimilarity between samples (sites; AD), per cent contribution (%), and cumulative per cent contribution (C%) of each helminth species to the overall pattern of similarity of component communities, landscape predictors (buildings, woodlands, fields, bare lands, and water) of species abundances at the 10 and 100 ha scales, parasite life cycle, and infection mode.

Taxon	AD	%	C%	Predictive landscape element(s)	Life cycle	Infection mode
*Strongyloides oswaldoi*	19.2	31.4	31.4	-	Monoxen	Infecting larva
*Microtetrameres pusilla*	8.1	13.1	44.5	10 ha: Woodlands^d^ (R^2^ = 35.9%, F_1,9_ = 5.032, *P* = 0.0497)	Heteroxen	Orthoptera
				100 ha: Buildings^i^ (R^2^ = 51.9%, F_1,9_ = 9.743, *P* = 0.0120)		
*Wardium fernandensis*	5.6	9.1	53.6	10 ha: Buildings^i^ (R^2^ = 36.4%, F_1,9_ = 5.139, *P* = 0.0478)	Heteroxen	Orthoptera/Coleoptera
				100 ha: Fields^d^ (R^2^ = 39.6%, F_1,9_ = 5.908, *P* = 0.0365)		
*Syngamus trachea*	5.1	8.3	61.9	-	Monoxen or Heteroxen	Ingestion of eggs or paratenic hosts (annelid)
*Conspicuum conspicuum*	3.6	5.9	67.8	10 ha: Fields^d^ (R^2^ = 46.2%, F_1,9_ = 7.722, *P* = 0.0207)	Heteroxen	Terrestrial mollusk and isopod
				100 ha: Fields^d^ (R^2^ = 51.6%, F_1,9_ = 9.613, *P* = 0.0124)		
*Lueheia inscripta*	3.3	5.4	73.2	-	Heteroxen	Blattodea (American cockroach)
*Dilepis undula*	3.3	5.4	78.6	-	Heteroxen	Annelid (earthworm)
*Lutztrema obliquum*	3.1	5.1	83.7	10 ha: Fields^d^, woodlands^d^, buildings^i^ (R^2^ = 79.5%, F_3,7_ = 9.092, *P* = 0.0088)	Heteroxen	Terrestrial mollusk and milliped
				100 ha: Woodlands^d^, fields^d^ (R^2^ = 83.8%, F_2,8_ = 20.715, *P* = 0.001)		
*Aproctella stoddardi*	2.5	4.1	87.7	10 ha: Buildings^i^ (R^2^ = 46.9%, F_1,9_ = 7.945, *P* = 0.0194)	Heteroxen	Mosquito vectors
				100 ha: Buildings^i^ (R^2^ = 53.1%, F_1,9_ = 10.189, *P* = 0.0108)		
*Fernandezia spinosissima*	2.3	3.8	91.5	10 ha: Bare lands^d^ (R^2^ = 36.4%, F_1,9_ = 5.139, *P* = 0.0478)	Heteroxen	Mollusks, annelids
				100 ha: Bare lands^d^ (R^2^ = 48.0%, F_1,9_ = 8.338, *P* = 0.0173)		
*Oxyspirura petrowi*	2.3	3.7	95.2	10 ha: Fields^d^ (R^2^ = 60.1%, F_1,9_ = 15.543, *P* = 0.0053)	Heteroxen	Arthropods (Blattodea)
				100 ha: Fields^d^ (R^2^ = 59.1%, F_1,9_ = 13.011, *P* = 0.0058)		
*Cardiofilaria* sp.	1.5	2.4	97.6	10 ha: Bare lands^d^ (R^2^ = 62.2%, F_1,9_ = 14.776, *P* = 0.0042)	Heteroxen	Mosquito vectors
				100 ha: Bare lands^d^ (R^2^ = 44.7%, F_1,9_ = 7.274, *P* = 0.0236)		
*Tamerlania inopina*	1.0	1.7	99.3	10 ha: Bare lands^d^ (R^2^ = 39.6%, F_1,9_ = 5.917, *P* = 0.0364)	Heteroxen	Terrestrial mollusk
*Aonchoteca* sp.	0.2	0.4	99.7	-	Heteroxen	Annelid (earthworm)
*Brachylaima* sp.	0.2	0.3	100	10 ha: Bare lands^d^ (R^2^ = 47.4%, F_1,9_ = 8.101, *P* = 0.0185)	Heteroxen	Terrestrial mollusk
				100 ha: Bare lands^d^ (R^2^ = 42.2%, F_1,9_ = 6.576, *P* = 0.0293)		

d direct relationship; ^i^ inverse relationship.

Whereas the abundance of *T. inopina* was predicted by the % bare lands only at the 10-ha scale, the abundances of nine helminth taxa were predicted by the availability of a single or a combination of landscape elements at both spatial scales. In most cases (7 of 9), the same landscape element(s) was(were) the best predictor(s) of abundance at both scales. In the only two exceptions (*M. pusilla* and *W. fernandensis*) the proportion of built-up areas was the best predictor at one scale, while another landscape element was the best predictor at the other scale. In all these cases the proportion of built-up areas showed a negative influence on parasite abundance, whereas the proportion of the other landscape element(s) showed a positive influence (Table 1). These results support hypothesis 4.

## Discussion

This study showed that urbanization disrupts host-parasite interactions. As expected, parasite species richness presented an inverse relationship with the degree of urbanization, but a direct relationship with environmental heterogeneity. Also, the structure of helminth communities differed along the rural-urban gradient and parasites responded differently to the availability of particular landscape elements. Finally, the similarity of the results at both spatial scales showed that the smallest, 10-ha scale was appropriate for identifying the influence of the landscape on the occurrence of helminth parasites on rufous-bellied thrushes along the gradient.

Our findings are compatible with the parasites' life cycles and the habitat requirements of their intermediate hosts and vectors. Additionally, the lack of influence of the availability of water on any helminth species is consistent with the terrestrial habits of thrushes [29], [43]. Several patterns emerged from the analyses of the influence of the representation of landscape elements on the abundance of species: (a) the abundance of both helminths that have a direct cycle (*S. oswaldoi* and *S. trachea*) was not predicted by any landscape element, (b) this pattern was also found for most species that have an annelid intermediate (*D. undula* and *Aonchoteca* sp.) or paratenic host (*S. trachea*), (c) bare lands and fields were the best predictors of the abundance of helminths that have molluscan intermediate hosts (*C. conspicuum*, *L. obliquum*, *F. spinosissima*, *T. inopina*, and *Brachylaima* sp.), (d) fields and woodlands were the best predictors of parasites using insect intermediate hosts (*M. pusilla*, *W. fernandensis*, and *O. petrowi*), and (e) both species transmitted by mosquito vectors were also affected by the landscape (*A. stoddardi* by % buildings and *Cardiofilaria* sp. by % bare lands; Table 1).

These patterns help to explain why the degree of urbanization was shown to be a good predictor of the spatial differences in parasite community structure along the rural-urban gradient. On the other hand, the lack of significant relationships between the abundance of most species that have an annelid in its cycle and the representation of landscape elements are likely to result from the fact that earthworms make up the largest animal biomass in the soil and are welcome at urban gardens because of the important ecosystem services that they provide [44]. These results are also consistent with the contention that green spaces may play an important role in biodiversity (including helminth) conservation in urban environments [45].

Urbanization may also increase the richness and abundance of some invertebrate intermediate hosts, like generalist coleopterans [46], isopods [47], and arachnids [48] (a decrease has been observed for other groups, orthopterans [49] and specialist coleopterans [46], hymenopterans [50], and isopods [47]) by, for example, bringing alien species that are associated with humans, such as cockroaches. Therefore, since host density plays a critical role in the dynamics of host-parasite interactions [51]–[54], the lack of any significant positive relationship between parasite abundance and % buildings may be related to a possible lower density of thrushes at predominantly built-up landscapes. An increase in the coverage of buildings (and aquatic environments) might impact thrush-parasite relationships by decreasing the amount and diversity of other landscape elements, thereby reducing the diversity and abundance of invertebrate intermediate hosts (and vectors) and the environment's carrying capacity for thrushes. Although environmental pollution may lower the efficiency of the immune system of hosts [55], the absence of influence of parasite richness on the weight of individual hosts (a proxy measure of health) [26] suggests that pollution would not be a good explanation for the proposed lower thrush density in more urbanized areas.

Future studies shall integrate population size assessments of definitive and intermediate hosts and vectors across rural-urban gradients for better understanding the influence of the urbanization process on host-parasite interactions. These studies shall also investigate hosts belonging to other guilds (*e.g.*, granivorous, nectarivorous, piscivorous, and scavengers) to provide us with a thorough view of the impacts of the replacement of natural environments with buildings on bird-parasite relationships.

## Supporting Information

Table S1 **Percentage urbanization at the 10-ha and the 100-ha scale, species richness, and abundance of each helminth species at the 11 study sites.**
(DOCX)
