# Supplementary material for: Urbanization Breaks Up Host-Parasite Interactions: A Case Study on Parasite Community Ecology of Rufous-Bellied Thrushes (Turdus rufiventris) along a Rural-Urban Gradient
Source: PLoS One. 2014 Jul 28;9(7):e103144. doi: 10.1371/journal.pone.0103144 (PMC4113371; doi:10.1371/journal.pone.0103144)
Supplement: Table S1 — Percentage urbanization at the 10-ha and the 100-ha scale, species richness, and abundance of each helminth species at the 11 study sites. (DOCX) [file pone.0103144.s001.docx]

Table S1. Percentage urbanization at the 10-ha and the 100-ha scale, species richness, and abundance of each helminth species at the 11 study sites.

| % | Bu | Species |  |  |  |  |  |  |  |  |  |  |  |  |  |  |  |
| --- | --- | --- | --- | --- | --- | --- | --- | --- | --- | --- | --- | --- | --- | --- | --- | --- | --- |
| 10-ha | 100-ha | richness | Brach | Tamer | Consp | Lutz | Fern | Dile | Ward | Lueh | Aonc | Oxys | Micro | Aproc | Card | Stron | Syng |
| 1 | 10 | 11 | 0 | 0 | 3.1 | 1.3 | 0 | 1.1 | 7.6 | 1.8 | 0 | 2.5 | 1.6 | 0.6 | 0.9 | 3.3 | 0.8 |
| 16 | 29 | 7 | 0 | 0 | 2.7 | 0.3 | 0 | 0 | 1.1 | 1.6 | 0 | 1.3 | 5.9 | 0.9 | 0 | 0 | 0 |
| 19 | 22 | 7 | 0 | 0 | 0.8 | 0.3 | 0 | 0.5 | 1.3 | 0 | 0 | 0 | 2.8 | 2.0 | 0 | 7.0 | 0 |
| 27 | 20 | 12 | 0 | 0 | 2.0 | 3.4 | 0 | 0.7 | 1.3 | 1.1 | 0.3 | 0.6 | 1.9 | 1.0 | 0.2 | 4.3 | 0.1 |
| 36 | 34 | 13 | 0.3 | 1.2 | 1.4 | 0 | 2.6 | 1.7 | 0.6 | 0.5 | 0.1 | 0 | 2.5 | 0.9 | 1.4 | 0.5 | 0.2 |
| 51 | 85 | 10 | 0 | 0 | 0.4 | 0.1 | 0 | 0.4 | 1.3 | 1.7 | 0 | 0.1 | 0.1 | 0.6 | 0.1 | 9.9 | 0 |
| 52 | 85 | 9 | 0 | 0.3 | 1.8 | 0 | 0.3 | 2.1 | 2.0 | 2.7 | 0 | 0 | 0.1 | 0 | 0 | 21.5 | 0.4 |
| 72 | 45 | 10 | 0 | 0 | 1.8 | 0 | 0.6 | 0.2 | 0.4 | 0.4 | 0 | 0 | 3.2 | 0.4 | 0.2 | 1.0 | 0.8 |
| 94 | 87 | 4 | 0 | 0 | 0.8 | 0 | 0 | 0.6 | 0 | 0.2 | 0 | 0 | 0 | 0 | 0 | 0.8 | 0 |
| 94 | 92 | 8 | 0 | 0 | 0.7 | 0.7 | 0.2 | 2.2 | 0.3 | 0.8 | 0 | 0 | 0 | 0 | 0 | 3.2 | 6.3 |
| 97 | 86 | 10 | 0 | 0.2 | 0.9 | 0.3 | 0 | 1.4 | 0.9 | 0.3 | 0 | 0 | 0.1 | 0.3 | 0 | 3.2 | 0.1 |

% Bu=% urbanization;

Digenea: Brach=*Brachylaima* sp.; Tamer=*Tamerlania inopina*; Consp=*Conspicuum conspicuum*; Lutz=*Lutztrema obliquum*;

Cestoda: Fern=*Fernandezia spinosissima*; Dile=*Dilepis undula*; Ward=*Wardium fernandensis*;

Acanthocephala: Lueh=*Lueheia inscripta*;

Nematoda: Aonch=*Aonchoteca* sp.; Oxys=*Oxyspirura petrowi*; Micro=*Microtetrameres pusilla*; Aproc=*Aproctella stoddardi*; Card=*Cardiofilaria* sp.; Stron=*Strongyloides oswaldoi*; Syng=*Syngamus trachea*.
